# Observations on the Fevers and Dysentery of Hot Climates; and on the Use of Mercury in Those Diseases

**Published:** 1793

**Authors:** William Boag

**Affiliations:** Surgeon in the Service of the Honourable East-India Company at Bombay.


### MEDICAL FACTS

AND

### OBSERVATIONS.

I. *Obſervations on the Fevers and Dyſentery of hot Climates; and on the Uſe of Mercury in thoſe Diſeaſes. By Mr.* William Boag, *Surgeon in the Service of the Honourable Eaſt-India Company at Bombay. Communicated in a Letter to* William Saunders, *M. D. Fellow of the College of Phyſicians, London, and Phyſician to Guy's Hoſpital; and by him to Dr.* Simmons.

*To Dr.* Saunders.

Dear Sir,

I HAVE intended for ſome time paſt to give you a ſhort account of the peculiarities I have obſerved in the nature and treatment of ſome of the diſeaſes of this country.

If there be any utility in what I ſhall relate, I know no one to whom I can addreſs myſelf ſo properly as to you, as, I believe, few can make their information upon any medical ſubject ſo generally uſeful.

I mean to confine my remarks chiefly to fevers and to dyſenteries, the two principal and moſt fatal diſtempers of this, in common with all other hot climates.

It will not be neceſſary to deſcribe the ſymptoms of thoſe diſeaſes, as this has been ſo often done already by different authors, particularly by Sir John Pringle, Dr. Hillary, and Dr. Cleghorn.

The fevers of hot climates have commonly been deſcribed under the names of bilious, remitting, yellow, or putrid fevers; but theſe terms are, for the moſt part, ill defined, and ſome of them ſeem to expreſs the ſtage or degree of fever, rather than the kind—Dr. Cleghorn obſerving, probably, the objections to which the more common appellations are liable, conſiders all the fevers of Minorca under the head of tertians. But though he claſſes them in this manner, he, at the ſame time, obſerves the great irregularities to which their paroxyſms are ſubject, how readily they aſſume a more more continued form, and how commonly they alternate with attacks of dyſentery. When they put on this irregular appearance they exactly conſtitute the bilious fevers of other authors, and it is to theſe fevers, by far the moſt common in this country, to which I mean to confine my remarks.

The ſymptoms of dyſentery are the ſame in all countries; the diſeaſe can therefore require no diſcrimination from me. But it will be neceſſary to make a few ſhort obſervations upon ſome theoretical opinions which have been entertained concerning the nature and cauſes of fevers and dyſenteries, in order that the principles may be underſtood upon which the practice hereafter deſcribed is founded.

The fevers of which we are ſpeaking, and dyſenteries, have always been obſerved to prevail in the ſame ſeaſons; and there is ſuch a general reſemblance of character betwixt them, that both ancient and modern authors have conſidered them as originating from the ſame cauſes. Though they differ much in their opinions reſpecting the nature of thoſe cauſes, yet this one point of a common origin being eſtabliſhed from ſuch united authority, is a ſtep of ſome importance in aſcertaining the nature of both.

The more ancient phyſicians, obſerving the great diſcharges of bilious matter in thoſe diſeaſes, believed that they originated from redundant or corrupted bile. This opinion was very generally adopted by their ſucceſſors, but it is now ſucceeded by the more laboured, though not more ſatisfactory, theories of modern date.

It would be ſuperfluous for me to give any account of thoſe theories; no one is better acquainted with their fallacy than you are: I will only beg leave to remark, that the total rejection of the ancient doctrine ſeems to be in ſome degree owing to writers in general not having had opportunities to obſerve the fevers and dyſenteries in warm climates, where they are infinitely more common than in Europe.

I am very far, however, from believing that bile alone, either redundant or corrupted, is ſufficient to produce thoſe fatal diſtempers; but it muſt alſo be confeſſed, that the bile cannot be in either of thoſe conditions, unleſs the organ which ſecretes this fluid be to a certain degree diſeaſed: for I hold it to be a fact as clear and demonſtrable as any in phyſic, that whenever a fluid is ſecreted by any gland in the body body different in quantity and quality from what is natural, the fault is not ſo much to be looked for in the fluid that is ſecreted, as in the organ that ſecretes it. The fluid may produce much diſorder in the ſyſtem, but the ſource of this diſorder is evidently in the gland.

If therefore it can be made to appear that the ſtate of the liver has ſome connexion with the production of thoſe diſeaſes, it will ſhow that although the ancients did not arrive at the truth, yet they approached towards it.

To illuſtrate this opinion, I ſhall beg leave to enumerate the following arguments:

1. In the fevers and dyſenteries of hot climates, large and ſpontaneous diſcharges of bile frequently happen, commonly to the great relief of the patient.

2. A pain, ſometimes dull, ſometimes acute, is, for the moſt part, felt by preſſing with the hand upon the region of the liver. As the diſeaſe advances this pain increaſes, and often a tumefaction and hardneſs become perceptible in that region. This obſervation is ſtrongly corroborated by Dr. Hillary, in his account of the putrid bilious fever of the iſland of Barbadoes.

"We likewiſe," ſays he, "obſerve, that "the hypochonders, eſpecially the right, and

" the adjoining præcordia, are moſt affected

" throughout the whole time of the diſeaſe,

" which is the ſeat of the liver and gall blad-

" der, inſomuch that Dr. Warren ſays, it

" ſeems to be the ſeat and throne of the diſ-

" eaſe; and I have always obſerved that the

" ſick cannot bear the leaſt preſſure of one's

" hand upon the parts where the gall bladder,

" and biliary ducts, and liver, are ſituated."

This pain, however, is not conſtantly to be diſcovered, even by the moſt careful examination: this may be owing to the inſenſibility of the liver, or to the diſeaſed part being beyond the reach of preſſure with the hand. But it is not uncommon for the diſeaſe of the liver to ſhow itſelf, in this country, by a fixed pain in the top of the ſhoulder, without any pain being felt in the organ itſelf.

3. In fevers a yellowneſs of the ſkin is often obſerved, as if the patient were afflicted with the jaundice; hence the name of yellow fever, ſo common in the Weſt Indies. But this ſymptom is as often obſerved in dyſentery as in fevers, and, I believe, it can be fairly aſcribed to no other cauſe than to the bile being carried into the circulation in conſequence of the diſeaſed ſtate of the liver.

4. If there be any reafon to believe that the liver is affected from the beginning, it will account in the moft rational manner for the enlargements in the fide fo often noticed by authors, after thofe difeafes have continued fo long as to become chronic.

5. The moft fatisfactory way of gaining information concerning the nature of any internal difeafe is by diffection of the dead fubject.

I have opened a number of bodies, and been prefent at the diffection of a great number more, of thofe who have died either of fever or of dyfentery in this ifland, where the cafes of the patients, previoufly to their deaths, were accurately taken, and the appearances on diffection carefully noted down.—All that I can attempt in this letter is to ftate, in a general way, the appearances obferved in thofe diffections.

In the cafes both of fever and dyfentery the liver was, with two exceptions, conftantly found difeafed.

In moft cafes it was much enlarged, fometimes indurated, but more frequently very foft, fo as to tear upon a flight touch.

Commonly an abfcefs had formed in it, fometimes of great extent, and fometimes fo

ſmall as only to be detected by a minute inſpection.

The diameter of the blood veſſels, through the whole ſubſtance of this viſcus, was commonly found much increaſed, and their coats proportionably thickened. They were alſo obſerved to be, for the moſt part, empty.

In two caſes of dyſentery where the patients had coughed up matter for ſome time before their death, a large abſceſs in the liver had made its way through the diaphragm into the lungs.

The gall bladder was ſometimes very much diſtended with yellow ropy bile.

The ſpleen was, in moſt inſtances, much enlarged, its texture looſened, and ſometimes totally deſtroyed; the ſubſtance remaining having no other appearance than that of a dark coagulum of blood. This was particularly the caſe in the two inſtances above mentioned, where no diſeaſe was apparent in the liver.

In ſome inſtances the pancreas was conſiderably enlarged and ſchirrous.

In patients who died of the dyſentery the bowels were conſtantly found much inflamed. In the worſt caſes, mortification had taken place, place, eſpecially in the rectum and part of the colon.

In dyſenteric patients alſo the meſenteric glands were commonly ſeen enlarged.

A degree of inflammation, more or leſs conſiderable, was uſually obſerved in the inferior portions of the lungs, contiguous to the diaphragm, and was commonly moſt remarkable on the right ſide of the cheſt.

In Dr. Hunter's Treatiſe on the Diſeaſes of the Army in Jamaica there is an account of the diſſections of twenty-three men who died of what is termed the bilious fever, and in every one of thoſe caſes it is ſaid that the liver was more or leſs diſeaſed.

From the whole of this ſtatement, I think it will be admitted, that the liver or ſpleen, or moſt probably both, ſuffer in the courſe of theſe diſeaſes; for from the mutual connexion which ſubſiſts betwixt thoſe organs, it is highly probable that the one cannot be diſeaſed without the ſympathy of the other: and the facts I have mentioned appear to me ſufficiently ſtrong to authorize this conjecture, "that a diſeaſed "ſtate of the liver and ſpleen has an intimate "connexion with the production of fevers and "dyſenteries in hot climates.'

It

It is, no doubt, difficult to underſtand how a diſeaſe of thoſe viſcera ſhould induce the paroxyſms of a fever, nor do I pretend to explain it; but there are many other phenomena of the animal œconomy equally inexplicable. We can ſometimes trace the cauſes of diſeaſes, but it is ſeldom in our power to aſcertain their mode of action.

In the caſe of dyſentery, however, there is not quite ſo much obſcurity.

If it be true that a dyſentery can only ariſe in conſequence of ſome ſtimulating matter being poured into the alimentary canal, and this ſeems to be actually the fact, notwithſtanding what has been ſaid to the contrary *, it is evident this matter can come but from few ſources. It may come from the ſtomach; it may be ſecreted by the veſſels on the internal ſurface of the inteſtines themſelves; or it may come from one or more of thoſe glands whoſe excretory ducts terminate in the duodenum. Of all theſe ſources vitiated bile, from a diſeaſed liver, ſeems the moſt remarkable: this, by continually diſcharging itſelf through the ductus communis

* Dr. Cullen's Firſt Lines of the Practice of Phyſic, Vol. III.

choledochus into the inteftines, will produce that conftant irritation, and all thofe other fymptoms, which accompany and characterize the dyfentery. Befides, the bowels being now deprived of healthy bile, which is abfolutely neceffary for the digeftion and affimilation of our food into chyle, will lofe their natural tone; lofs of appetite and indigeftion muft enfue; and the contents of the ftomach, inftead of affording nourifhment to the body, will become a frefh fource of uneafinefs and diforder.

The juftnefs of thefe obfervations did not efcape the penetration of Dr. Cullen, who makes it a queftion, how far the ftate of the bile may be connected with the production of dyfentery.

Upon exactly fimilar principles it may be afferted, that a difeafed pancreas may produce a dyfentery; and, I think, there is great reafon to believe that it fometimes does; but probably not fo often as a difeafed liver.

Some authors have thought that obftructed mefenteric glands are a caufe of dyfentery, and the appearance of thofe glands on diffection would feem to countenance this opinion; but I apprehend it is rather an effect than a caufe of the difeafe. The extremities of the lacteals being irritated by the vitiated matter in the inteftines, will will produce an obſtruction and inflammation in the glands of the meſentery, as happens with every other lymphatic gland of the body under like circumſtances.

With reſpect to the exciting cauſes of the fevers and dyſenteries I have little to obſerve. Thoſe commonly enumerated are, putrid effluvia, obſtructed perſpiration, intemperance, &c. Theſe, I make no doubt, have conſiderable influence; but theſe diſeaſes are ſo conſtantly prevalent in this country, that they muſt depend on ſome cauſe equally conſtant with themſelves: this I believe to be the heat of the climate.

It is certain that the bile is more abundantly ſecreted, and the liver more frequently diſeaſed, in warm latitudes than in Europe. This remark extends with equal truth to the brute creation; the livers of our domeſtic animals being frequently found after death in a ſtate of ſuppuration.

Beſides, it is an obſervation agreeable to the experience of all authors, that the diſeaſes of which I am ſpeaking, never make their appearance in Europe but in the hot months of the year; hence it is more than probable that the ſtate of the atmoſphere has great influence in producing them.

Moſt writers have aſſerted that theſe diſeaſes are of a very contagious nature; Dr. Cullen is ſo ſtrongly prepoſſeſſed with this opinion, in the caſe of dyſentery particularly, that in defining this diſeaſe, he has choſen to make its contagious nature a part of his definition.

I am, however, very doubtful how far this opinion may be well founded; I never knew or heard of an inſtance of either of theſe diſeaſes running through a family in the manner highly contagious diſeaſes commonly do; nor does it by any means appear that the attendants on the ſick are peculiarly liable to be affected with them. As this, however, is a point not always to be eaſily aſcertained, I will not take upon me to ſay that they are not in any caſe, or in any ſituation, contagious. But it may here be obſerved, that this climate does not ſeem favourable either to the production or propagation of infectious diſeaſes. A remarkable inſtance of this we have in the caſe of ſmall pox: theſe commonly prevail here during the cold months of the year, but gradually diſappear as the hot ſeaſon advances.

Much of what I have hitherto ſaid muſt, no doubt, be conſidered as matter of ſpeculation; but in ſpeaking of the practice, I ſhall lay aſide all all theory, and merely ſtate what I know from my own obſervation, aſſiſted alſo with the experience of others.

At the beginning both of fevers and of dyſenteries, the firſt thing directed is, to evacuate the contents of the alimentary canal by mild remedies, and during their progreſs to obſerve that the bowels are always kept in a ſoluble ſtate. This practice is particularly recommended by Dr. Sydenham and by Sir John Pringle; and coming from ſuch authorities it can require little other recommendation.

After moderate evacuations, if the fever be mild, and eſpecially if it be attended with conſiderable remiſſions, the bark will often be ſufficient to accompliſh a cure.

If, on the contrary, it puts on a more threatening aſpect, or has been of ſome continuance, the bark, though it may be uſed as an auxiliary, ought not alone to be depended on: the medicine which in this caſe has been found, from much experience, moſt certain and efficacious is mercury.

In dyſentery alſo, if the complaint ſhould not yield to ſimilar evacuations, followed by a moderate uſe of opium, the ſame remedy ought to be had recourſe to.

Suppoſing

Suppoſing then, that the uſe of mercury be judged neceſſary, which moſt frequently happens, it may be proper to make a few ſhort obſervations on the manner of uſing it, and the effects it produces in theſe diſeaſes.

1ſt. It has been found, though I do not pretend to explain the fact, that patients in this country generally require a greater quantity of mercury to produce its uſual effects upon the ſyſtem than in Europe.

2dly. As the liver, both in the fevers and dyſenteries, is apt to run very unexpectedly into a ſtate of ſuppuration, it is of much conſequence that the exhibition of the mercury be carried to the neceſſary length as ſoon as poſſible.

The manner, therefore, of uſing mercury in thoſe complaints in the general hoſpital here, is, to rub two drachms of the ſtrong mercurial ointment upon the belly, legs, and thighs of the patient, every night and morning, till it ſenſibly affects the mouth, which commonly happens in three, four, or five days; when the patient will, for the moſt part, find himſelf greatly relieved: and if the ointment be continued for a day or two more, no complaint will remain but a ſore mouth.

If this quantity be not ſufficient to affect the mouth ſpeedily, it is either increaſed, or the internal uſe of ſome mild preparation of mercury is joined to the external friction; in this way as much is done in a few days as could be done in a fortnight by following the common method of giving mercury in Europe.

It happens more frequently than might be expected, that it is either very difficult to affect the mouth, or that it cannot be done at all, by the uſe of mercury in any form.

Whether this depends upon a particular diſeaſed condition of the lymphatic ſyſtem, or upon the peculiar conſtitution of the patient, is not eaſy to determine: but upon whatever cauſe it may depend, it is a moſt unfavourable circumſtance; ſo much ſo, that I do not recollect ever to have ſeen a patient recover whoſe mouth could not be touched by the mercury.

I have ſeen the mercurial ointment employed in the moſt free manner in the advanced ſtage of a fever and of a dyſentery, and apparently ſnatch the patient from the grave. It is true I have alſo ſeen it fail: the medicine is by no means infallible; but it ſeems to be by far the moſt efficacious remedy yet diſcovered for the cure of theſe diſeaſes. Upon the whole, it would appear that the the due action of this remedy is ſo much connected with the ſpeedy recovery of the patient, that I think it may be ſtated as a general rule, " that the uſe of mercury ſhould be " urged with a vigour ſomewhat proportioned " to the urgency of the complaint."

It would carry me far beyond my purpoſe to mention the different remedies recommended by authors for the relief of particular ſymptoms :—probably the uſe of mercury may reduce them within a ſmaller compaſs. I would take the liberty of making only one obſervation, with reſpect to the uſe of opium in the dyſentery. This medicine is no doubt very neceſſary in that complaint, but it is always injurious if carried ſo far as ſuddenly to check the flux. Here it may be uſeful to recollect an old maxim in phyſic, that " phyſicians are " not the directors, but the miniſters of na" ture ;"—the diſcharge is to a certain degree a ſalutary effort, and cannot with ſafety be altogether reſtrained.

From the view which we have taken of this diſeaſe, it will be evident, that the uſe of aſtringents, eſpecially at the beginning, is highly improper. But their uſe ſeems to have been for ſome time paſt rejected by practitioners, though upon different principles *.

Relapſes, both in fevers and dyſenteries, are not uncommon. I believe they are frequently brought on by imprudent expoſure to the ſun, or by ſome irregularities in diet.

So far as my experience goes, in a public hoſpital, relapſes prove often fatal to the patients. But this obſervation does not apply to private practice, where the ſucceſs is much greater.

It appears that a relapſe may often be prevented by giving ſmall and repeated doſes of mercury, till the patient recovers ſufficient ſtrength to take exerciſe.

It would be unneceſſary to ſay any thing of the treatment of fevers, when they aſſume a pure intermittent form, as they generally yield to the uſe of the Bark. When, however, they reſiſt this medicine, I have repeatedly known the combined uſe of bark and mercury produce the moſt beneficial effects.

The practice which I have thus briefly endeavoured to deſcribe, may, I make no doubt, be

* Cullen's Materia Medica, vol. ii. chap. i.

conconfidered by many as bold and hazardous; efpecially by thofe who believe that mercury putrifies the fluids, and diffolves the blood. Thefe notions are particularly inculcated by fome writers on the difeafes of warm climates; but fo far as I have been able to remark, in a very extenfive ufe of this medicine, they feem rather to have been formed in the clofet, than drawn from obfervation. Mercury, in all countries, is a dangerous remedy in the hands of the unfkilful, but may be both fafe and efficacious when ufed with prudence and judgment.

No man can form a true conception how far the ufe of mercury extends in a warm climate, unlefs he practife in it, and will fairly try its effects.

I have feen patients labouring under afthma and the moft obftinate coughs, after having ufed in vain every medicine generally recommended in fuch cafes, at length completely cured by the free ufe of mercury; thus proving in a fatisfactory manner, that the difeafe was feated in the liver, and that the affection of the lungs was merely fymptomatic.

I have only further to remark, that the method of treatment of which I have given you a very imperfect outline, is by no means new. It was the practice of Doctor Paisley, late physician-general at Madras, who was well known over most parts of India, as a man of the first professional abilities. Mr. Scott has contributed to introduce it into Bombay, and has applied it with great success in a very extensive practice. Nor can I conclude this letter without acknowledging my obligations to Mr. Scott, and to Mr. Stewart, both surgeons belonging to this presidency, for an early acquaintance with the diseases of this country.

You will therefore perceive, that if this mode of treatment be entitled to any praise, I claim no share in it.—I have rather related the improvements of others, than my own.

——— *Quæ non fecimus ipsi,*

*Vix ea nostra voco.*

I am, my dear Sir,

With the greatest respect and regard,

Your ever obliged Servant,

William Boag.

*Bombay,*

*January* 16, 1792.

